# Mental health problems, low economic capability, and social marginalization among 28,047 adults

**DOI:** 10.1093/eurpub/ckac129.614

**Published:** 2022-10-25

**Authors:** SH Haugland, A Topor, JG Friesinger

**Affiliations:** Department of Psychosocial Health, University of Agder, Grimstad, Norway

## Abstract

**Background:**

Mental health problems and low economic capability increase the risk of social marginalization. However, few larger studies with a broad age group have investigated how the cumulative effect of having both these difficulties may affect social life. The purpose of this study was to examine the associations between having mental health problems or having low economic capability or reporting both, and social marginalization.

**Methods:**

The study is based on responses from 28 047 adults (> 18 years old) from the general population participating in The Norwegian Counties Public Health Survey 2019. The study had a cross-sectional design and included respondents’ evaluation of own economic capability, mental health problems (HSCL-5) and social marginalization measured as lack of social support, low participation in organized social activities, low participation in other activities, missing someone to be with, feeling excluded and feeling isolated. Multivariable logistic regression was adjusted for sex and age.

**Results:**

Findings showed that having mental health problems or low economic capability were both associated with various measures of social marginalization with odds ratio [OR] 95% confidence interval [CI] ranging from OR 1.33; CI 1.23-1.43 to OR 12.63; CI 10.90-14.64. However, the odds of social marginalization strongly increased for respondents having both mental health problems and low economic capability compared to respondents without both these difficulties, with OR ranging from OR 2.08; CI 1.90-2.27 to OR 29.46; CI 25.32-34.27. Findings are preliminary.

**Conclusions:**

Having both mental health problems and low economic capability strongly increases the risk of social marginalization, far beyond the effect of each factor alone.

**Key messages:**

• Having both mental health problems and low economic capability strongly increases the risk of social marginalization, far beyond the effect of each factor alone.

• Services should aim to develop multi-faceted approaches to meet multiple life-challenges to prevent social marginalization.

